# 

**Published:** 1817-07

**Authors:** 


### THE LONDON MEDICAL AND PHYSICAL JOURNAL;

CONTAINING

ORIGINAL CORRESPONDENCE of EMINENT PRACTITIONERS,

AND THE

EARLIEST INFORMATION ON SUBJECTS CONNECTED WITH MEDICINE, SURGERY, CHEMISTRY, PHARMACY, BOTANY, AND NATURAL HISTORY.

---

*Successively conducted by*

T. BRADLEY, M.D.

A. F. M. WILLICH, M.D.
R. BATTY, M.D.
A. A. NOEHDEN, M.D.
J. ARNEMAN, M.D.
J. P. PFAFF, M.D.
J. ADAMS, M.D.
W. SHEARMAN, M.D.
W. ROYSTON, Esq.

AND

SAMUEL FOTHERGILL, M.D.

Assisted by eminent Gentlemen, in the various Branches of the Profession.

---

VOL. XXXVIII.

FROM JULY TO DECEMBER, 1817.

ARMY MEDICAL LIBRARY
5144
WASH[illegible] D.C., U.S.A.

---

Et quoniam variant morbi, variabimus artes;

Mille mali species, mille salutis erunt.

---

*LONDON:*

PRINTED FOR THE PROPRIETORS, BY J. ADLARD, 23, BARTHOLOMEW-CLOSE, AND 39, DUKE-STREET, SMITHFIELD.

PUBLISHED BY J. SOUTER, NO. 73, ST. PAUL'S CHURCH-YARD; AND MAY BE HAD OF ALL BOOKSELLERS.

W1
LO 31

Dr. MERRIMAN and Dr. LEY will re-commence their Lectures on Midwifery, and the Diseases of Women and Children, at the Middlesex Hospital, on Monday, July 7th, at half-past ten o'clock.

---

Dr. SCUDAMORE has in the press, and nearly ready for publication, (the second edition, corrected and enlarged,) a Treatise on the Nature and Cure of Gout and Rheumatism; including general considerations on Morbid States of the Digestive Organs, some remarks on Regimen, and practical observations on Gravel.

In the press, the Principles of Diagnosis, by MARSHALL HALL, M.D. &c. This work is founded entirely on the External Appearances of Morbid Affections. It embraces—1. A view of the Countenance and Attitude of Patients, inasmuch as they are plainly characteristic of diseases. 2. The Symptoms of Diseases, considered in their modifications, and in relation to particular affections. 3. A Diagnostic Arrangement of Diseases. And, lastly, their Diagnosis. —A part of this work will be published in July.

Speedily will be published, Vol. I. of the Dublin Hospital Reports and Communications in Medicine and Surgery. The work will consist of two parts:—1st. Annual Reports from Medical and Surgical Hospitals; 2d. Miscellaneous Communications on Medical and Surgical Diseases, tending to the improvement of pathology and practice. The work will be edited by four physicians or surgeons to extensive hospitals, who will publish one volume every year.

Mr. BAYFIELD, Surgeon-Cupper to Guy's Hospital, intends shortly to publish a concise Treatise on the Art of Cupping. The work will be illustrated by Cases.

Shortly will be published, in two volumes, octavo, a Translation of Professor Orfila's Elementary Treatise on the Science of Chemistry: it is expected that this work will include all the modern chemical discoveries which have been made here and in France; and, as such, will be peculiarly interesting to the student.

---

### *NOTICES TO CORRESPONDENTS.*

*The length of our Retrospect has obliged us to defer the insertion of Communications from* Dr. KINGLAKE, Mr. KER, Mr. BISH, ANIMICUS, *&c. besides many foreign correspondents, which shall appear in our next.*

*We are obliged to* AMICUS *for the trouble he has taken in explanation.*

